# Panhypopituitarism in an Elderly Patient: A Case Report of Pituitary Macroadenoma Presenting With Sepsis-Like Symptoms

**DOI:** 10.7759/cureus.92168

**Published:** 2025-09-12

**Authors:** Muhammad Dalili, Shadman Sakib Rahman, Sumaiya Kamal, Zarshal Zakir

**Affiliations:** 1 Diabetes and Endocrinology, Medway Maritime Hospital, Gillingham, GBR; 2 Internal Medicine, Medway NHS Foundation Trust, Gillingham, GBR; 3 Acute Medicine, Medway Maritime Hospital, Gillingham, GBR; 4 Medicine, CHI St. Alexius Health, Bismarck, USA

**Keywords:** elderly population, hypogonadism, macroadenoma, panhypopituitarism, thyroid hormone replacement

## Abstract

Panhypopituitarism is uncommon but clinically significant, most often caused by pituitary macroadenomas. Its presentation can be subtle and nonspecific, which may lead to misdiagnosis, particularly in elderly patients. We report the case of an 85-year-old woman admitted with fever, abdominal pain, hypotension, and acute confusion, initially managed as sepsis. Laboratory tests revealed hyponatremia and multiple anterior pituitary hormone deficiencies. Magnetic Resonance Imaging (MRI) confirmed a pituitary macroadenoma with suprasellar extension. The patient showed rapid clinical improvement after initiation of hydrocortisone and levothyroxine replacement. Ophthalmologic evaluation demonstrated preserved visual function, supporting conservative management. This case underscores the importance of considering endocrine causes, especially adrenal insufficiency due to pituitary disease, in elderly patients presenting with sepsis-like symptoms. Early recognition and timely hormone replacement can be life-saving.

## Introduction

Panhypopituitarism is the loss of multiple anterior pituitary hormones. It may result from tumors, ischemia, inflammation, or infiltrative disease [1]. Pituitary macroadenomas, defined as lesions over 10 mm, are the most common cause [2]. Panhypopituitarism from a pituitary macroadenoma usually develops gradually and combines features of hormone deficiency with those of a mass lesion. Symptoms depend on which hormones are missing but often include adrenal insufficiency, hypothyroidism, hypogonadism, and growth hormone deficiency [3].

Patients may have fatigue, weight gain, cold intolerance, low libido, infertility, amenorrhea, or loss of body hair due to reduced adrenocorticotropic hormone (ACTH), thyroid-stimulating hormone (TSH), luteinizing hormone (LH)/follicle-stimulating hormone (FSH), growth hormone (GH), and prolactin function. Headache and visual field defects, especially bitemporal hemianopia from optic chiasm compression, are common with large tumors. In some cases, acute hemorrhage or infarction of the macroadenoma, also known as pituitary apoplexy, causes sudden severe headache, vision loss, ophthalmoplegia, and acute adrenal crisis [3].

In older patients, signs are often nonspecific and may resemble infection or metabolic disease [4]. Delay in diagnosis increases risk, especially if adrenal insufficiency is untreated. This case highlights the value of early suspicion and endocrine testing in patients with atypical presentations.

## Case presentation

An 85-year-old woman with chronic kidney disease, hypertension, diabetes, Lynch syndrome, and prior nephrectomy presented with two days of fever, abdominal pain, vomiting, dysuria, and shortness of breath. She also reported constant right groin pain radiating to the back. Her diabetes had been well controlled, with hemoglobin A1c (HbA1c) values of 6.8% on admission. Kidney function had remained stable over recent years, with a baseline estimated glomerular filtration rate (eGFR) of 60 in January 2020 and a gradual decline to 45-50 during the past year; her eGFR on admission was at this baseline. Blood pressure measurements taken regularly at her general practitioner (GP) regularly between 2020 and 2025 were consistently well controlled, with systolic values ranging 115-125 mmHg and diastolic values ranging 70-85 mmHg.

On admission she was hypotensive (88/60 mmHg), dehydrated, and needed oxygen, with initial room air saturations of 92-93% in the emergency department (ED). She required 2 L supplemental oxygen to maintain saturations above 94% and remained stable around 96% on this support. Temperature was 37.7°C. Abdominal exam revealed right-sided nephrectomy scar, soft abdomen with epigastric and right lower quadrant tenderness, rebound tenderness, and present bowel sounds. Cardiovascular exam showed normal heart sounds with no added murmurs. Chest was clear on auscultation, and a chest X-ray performed the following day showed no consolidation, collapse, or significant pleural effusion, with an unremarkable cardiomediastinal silhouette. Neurological exam was normal, with no focal deficits and a Glasgow Coma Scale (GCS) of 15/15.

Initial laboratory investigations are shown in Table 1.

**Table 1:** Routine Blood Results WCC: white cell count, CRP: C-reactive protein, ALP: alkaline phosphatase, ALT: alanine aminotransferase, GFR: glomerular filtration rate

Test	Result	Normal Range
WCC (×10⁹/L)	14.2	4.0 – 11.0
Hemoglobin (g/dL)	163	130 – 170
Neutrophils (×10⁹/L)	10.9	2.0 – 7.0
CRP (mg/dL)	3.2	0.0 – 5.0
Sodium (mmol/L)	128	133 – 146
Potassium (mmol/L)	3.6	3.5 – 5.3
ALT (U/L)	97	< 35
ALP (U/L)	134	30 – 130
Bilirubin (µmol/L)	24	0 – 21
Estimate GFR (IU/L)	47	28-100
Creatinine (umol/L)	98	45-84
Albumin (g/L)	45	35-50
Urea (mmol/L)	4.6	2.5-7.8

**Figure 1:**
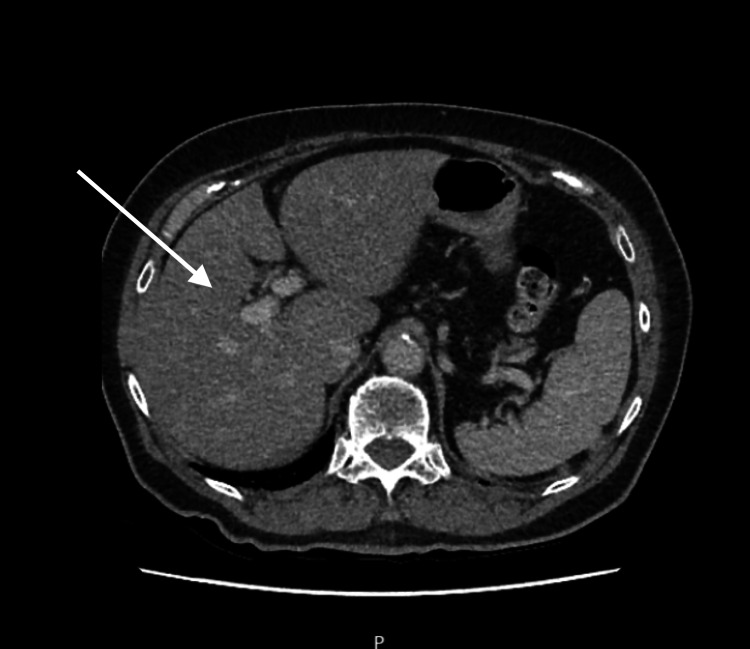
CT Abdomen and Pelvis with Contrast (CTAP): Post-Nephrectomy Findings Contrast-enhanced CT abdomen and pelvis demonstrates no acute intra-abdominal pathology. Postsurgical changes consistent with prior right nephrectomy are noted.

CT abdomen/pelvis showed no acute findings, aside from prior nephrectomy (Figure 1).

The patient was started on IV fluids, antibiotics, and oxygen. She initially received a stat dose of gentamicin along with ciprofloxacin for two days to cover both upper respiratory infection (URI) and presumed lower respiratory tract infection (LRTI). A urine culture taken on admission was negative; microscopy showed WBC 5/cumm, RBC 1/cumm, and epithelial cells 1/cumm, with culture demonstrating no significant growth (<10^5 cfu/ml). After discussion with the microbiology consultant, ciprofloxacin was discontinued and clarithromycin was started in its place. Viral swabs were also negative for COVID-19, influenza A, influenza B and respiratory syncytial virus (RSV). Chest X-ray later showed left basal consolidation. She became acutely confused.

Hormone testing is given in Table 2. Findings confirmed panhypopituitarism.

**Table 2:** Hormone testing TSH: thyroid-stimulating hormone, IGF-1: insulin-like growth factor 1

Test	Result	Reference Range
TSH (mIU/L)	0.09	0.30 – 4.80
Free T3 (pmol/L)	3.6	4.2 – 6.9
Free T4 (pmol/L)	11.8	7.7 – 20.6
Random Cortisol (nmol/L)	29	—
Luteinizing Hormone (IU/L)	< 0.2	—
Follicle-Stimulating Hormone (IU/L)	1.4	—
Estradiol (pmol/L)	< 55	—
Prolactin (mIU/L)	605	—
IGF-1 (nmol/L)	4.1	4 – 22.8

**Figure 2:**
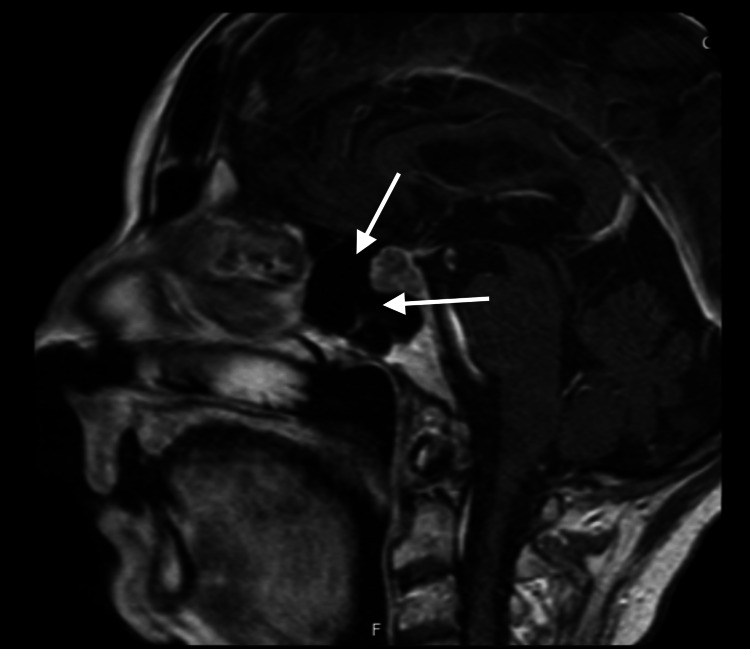
MRI Brain (Coronal section) MRI head showed a macroadenoma (pituitary macroadenoma). It is abutting optic chiasma. It measures length 13.7 mm x height 12.8 mm x breadth 18 mm. The pituitary stalk is thickened. The tuber cinereum is imaged normally.

MRI brain revealed a pituitary macroadenoma (13.7 × 12.8 × 18 mm) with suprasellar extension and compression of the optic chiasm (Figure 2).

The patient was started on hydrocortisone (20 mg in the morning, 10 mg at midday, and 10 mg in the evening) and levothyroxine (25 mcg daily). Within days, her confusion resolved and alertness improved. Ophthalmology review showed full ocular motility with no relative afferent pupillary defect, normal optic discs and retinal architecture on fundoscopy and optical coherence tomography (OCT), and mildly reduced color vision bilaterally (13/17 Ishihara plates). Visual field testing was unreliable but appeared grossly intact, indicating no significant optic chiasmal involvement (Table 3).

**Table 3:** Comprehensive Ocular Assessment Ophthalmology assessment showed preserved visual function. Motility was full with no RAPD. Discs and retinal architecture were normal. Color vision was mildly reduced bilaterally. Visual fields were unreliable but appeared intact. Visual acuity was normal in the right eye and corrected to normal in the left with pinhole, confirming refractive error rather than optic nerve involvement. Intraocular pressures were low-normal at 8 mmHg bilaterally, without evidence of ocular hypotony. Overall, findings supported conservative management with no indication of optic neuropathy. RAPD: relative afferent pupillary defect, OCT: optical coherence tomography

Investigation	Right Eye	Left Eye	Findings
Ocular Motility	Full	Full	No restriction
RAPD	Absent	Absent	Normal response
Fundoscopy / OCT	Normal	Normal	Normal discs and retinal architecture
Color Vision (Ishihara)	13/17	13/17	Mild bilateral reduction
Visual Fields	Unreliable	Unreliable	Grossly intact
Visual Acuity	6/6-3	6/9-2 (improves to 6/6 with pinhole)	Essentially normal; left eye deficit due to refractive error
Intraocular Pressure	8 mmHg	8 mmHg	Slightly below average, but safe and not pathologic

The patient’s eye examination showed essentially preserved visual function. Overall, the findings indicated no significant optic nerve compromise, supporting the multidisciplinary team’s decision for conservative management rather than urgent surgical intervention. Patient is stable at four months follow-up.

## Discussion

This case shows how panhypopituitarism can mimic sepsis in elderly patients. Fever, confusion, hyponatremia, and hypotension are common in infection but can also indicate adrenal crisis or pituitary failure [4]. Missing the diagnosis risks death.

Patient's labs confirmed adrenal insufficiency, central hypothyroidism, and hypogonadism. Low insulin-like growth factor 1 (IGF-1) showed growth hormone deficiency. The mild prolactin rise was likely stalk effect, not prolactinoma. MRI confirmed a macroadenoma with suprasellar extension, a typical cause of hypopituitarism [5].

Hormone replacement improved her condition quickly. Hydrocortisone was given before levothyroxine to avoid adrenal crisis, which follows standard management [6]. Ophthalmology confirmed no optic nerve damage, so surgery was not needed.

The differential diagnosis of hypopituitarism in this context is broad. In addition to pituitary macroadenoma, other structural and non-structural causes should be considered. Empty sella syndrome may present with partial or complete pituitary hormone deficiency and is often found incidentally on imaging. Pituitary apoplexy, either spontaneous or triggered by anticoagulation, can cause acute pituitary failure with severe headache and visual loss [7].

Furthermore, infiltrative disorders such as histiocytosis, sarcoidosis or hemochromatosis may damage the pituitary gland. Radiation-induced hypopituitarism or prior pituitary surgery are important historical clues. Less commonly, autoimmune hypophysitis, especially in patients with other autoimmune conditions, or metastatic disease to the pituitary, can mimic this presentation. Distinguishing between these requires careful clinical assessment, endocrine testing, and imaging [7].

This case underlines the need to test endocrine function in elderly patients with unexplained hyponatremia, confusion, or decline. Hypopituitarism remains underdiagnosed in this age group [8]. Awareness among clinicians can improve detection and outcomes.

## Conclusions

Panhypopituitarism from pituitary macroadenoma can look like sepsis in elderly patients. Hormonal testing and imaging are key for diagnosis. Early hormone replacement is life-saving and should be considered in unexplained acute illness with electrolyte disturbance or altered mental status.
